# Medical Wards of Mercy Hospital

**Published:** 1872-02-01

**Authors:** N. S. Davis


### Clinical Reports.

### MEDICAL WARDS OF MERCY HOSPITAL.

SERVICE OF PROF. N. S. DAVIS.

Case I. Valvular Disease of the Heart.—This patient entered the hospital three weeks ago, with much more embarrassment of respiration and irregularity of the heart's action than he now presents. There was considerable venous congestion of the lungs and oppression of breathing, and the heart's action was quick and tremulous, omitting one in four or five beats. Such symptoms may come from irregularity of innervation, from the cardiac ganglia of the sympathetic, or from disease at the base of the brain influencing the cardiac branches of the pneumogastric. But if the affection be organic a rough murmur will remain after the heart's action has been made slow and steady by digitalis, or other sedatives, and its location will determine the situation of the trouble. (Here each member of the hospital class was permitted to examine the cardiac region with the stethescope, and also with the ear direct.)

A double murmur is due generally to regurgitation; but sometimes it depends upon aortic and mitral lesion combined.

In this patient the rough sound is heard near the apex of the heart, and on its left side one inch below the nipple, and is rough, following the systole and seeming a continuation of it. It is plainly caused by mitral thickening, which, by the patient's account, was probably brought on by endocardial inflammation of a rheumatic character.

The great majority of this class of cases have their origin in rheumatic endocarditis. When of long standing they are generally incurable, but much may be done to palliate the patient's condition and prolong life. Whatever will lessen the frequency of the systolic action of the heart, and thereby give more time for the blood to pass through the contracted auriculo-ventricular opening, will afford temporary relief.

The patient was first put upon tincture digitalis, gtts. xx, every four hours, and to allay some gastric irritability and vomiting, which existed at the time of admission, he had carbolic acid and camph. tinct. opii, which speedily relieved that symptom. In two or three days the digitalis had brought the heart's action down to fifty beats per minute, and began to give a sense of tightness around the chest. The dose was then diminished to fifteen, and finally to twelve drops every six hours, which appears to be sufficient to keep the pulse beats between sixty and seventy per minute, though not regular. The digitalis has the advantage over other arterial sedatives of rendering the heart's action slow without diminishing its muscular force. As the patient was moderately anæmic, after the irritability of the stomach had been removed, he was directed to have twenty drops of the syrup of iodide of iron after each meal, moderate exercise in the open air, whenever it is pleasant, and the patient should study deliberation in all his movements. Thus he will have a pretty good lease of life. He has already improved greatly, and when quiet he feels nearly well.

Case II. Tuberculosis.—This patient, a laboring man aged about 38 years, says he has coughed more or less since the early part of last summer. He is considerably emaciated; has moderate night sweats; accelerated pulse; short respiration, and expectoration of mucopurulent matter. He has had no turns of hemoptysis. Physical examination of the chest, in which the class participated, showed flattening of the infra-clavicular regions; increased vibration of voice at the apex of the lungs; enfeebled inspiratory and prolonged expiratory murmur, anteriorly, with no rhonchus and no cavernous sounds. It was remarked that these physical signs indicated only primary or crude tuberculization of the upper lobes of the lungs, while the general symptoms of the patient indicated a more advanced stage of the disease. It was further remarked that if the physical examination was extended to the posterior part of the chest this apparent discrepancy between the general symptoms and physical signs would be explained by finding behind the upper part of the scapulæ the rhonchus indicating the stage of softening tubercles with inflammatory engorgement of the surrounding lung tissues. The case illustrates the necessity of making physical exploration of the whole chest. The Professor also related another case, in which a very important mistake was made by limiting the examination of the patient to the anterior part of the chest. The treatment should be such as is calculated to sustain nutrition and allay the local irritation in the lung tissue. In this case the patient was directed to take the following prescription:

℞ Comp. Syrup Squills ℥ jss.

Tinct. Bloodroot ℥ ss.

Camp. Tinct. Opii ℥ ii.

Mix. Take one teaspoonful each morning and evening.

℞ Syrup Iodide of Iron ℥ i.

Glycerine (pure) ℥ iii.

Mix. Take one teaspoonful after each meal time.

Case III. Splenitis and Chronic Ague.—This patient was admitted to the hospital two days since. He is a native of Ireland, aged about 30 years, and has been working in a district of country where periodical fevers are of frequent occurrence. He states that he has had paroxysms of intermittent fever every alternate day, with occasional interruptions, for a week, during the last three months. He has had one paroxysm, consisting of a well marked chill and fever, since he came into the hospital. His skin and lips are pale, indicating an impoverished or spanemic state of the the blood; but the most prominent item of complaint, on the part of the patient, is a severe pain in the left hypochondriac region, aggravated by a short, dry cough, with loss of appetite and general muscular weakness. On making physical examination, the respiratory murmur was found rough and exaggerated, indicating slight bronchitis; the left hypochondriac region was fuller than the right; decided tenderness on percussion at the lower margin of the ribs, with dullness extending vertically from two inches above the margin of the ribs to the crest of the left ilium; and transversely from the left margin of the epigastric region to the spine. With the patient on his back, and the thighs flexed on the pelvis, the members of the class could trace, by the touch, the hard rounded edge of a solid tumor extending from the margin of the epigastric region downward and to the left until it nearly touched the crest of the ilium. Both percussion and the touch showed that the broadest part of the tumor was upward and extended fully under the ribs of the left side. These facts showed that the tumor was simply an enlarged spleen. After pointing out the diagnostic differences between enlargements of the spleen, kidneys, mesenteric glands and ovaries, the Professor remarked that the moderate degree of tenderness existing in the spleen, the recent date of the enlargement, and the fact that it co-existed with chronic ague rendered it probable that it was caused by simple inflammatory congestion with semi-plastic exudation into the texture of the organ.

The treatment in such cases should have for its objects both the permanent arrest of the intermittent paroxysms and the removal of the local inflammation. If the former is not accomplished each chill and febrile exacerbation will renew the local congestion and defeat all treatment directed to that alone. The patient was directed to have five grains of sulphate of quinine with two of blue mass each morning and noon for two days, then the quinine alone every morning for a week or more, at the same time he was to take ten grains of hydrochlorate of ammonia, dissolved in syrup of liquorice, every four hours until the splenitic enlargement disappeared—the latter was stated to be an old remedy for visceral enlargements consequent on chronic ague, it having been recommended by Dr. Eberle in his work on practice. After the intermittent paroxysms have ceased, and the local symptoms of inflammation have abated or disappeared, it will be proper to keep the patient for some time on small doses of quinine in combination with a soluble salt of iron, of which the citrate and phosphate are the best. This combination should be continued, with a mild, easily digested diet, and moderate out of doors exercise, until the patient regains his muscular vigor, and the blood its natural proportion of red corpuscles.

CASE IV. CHRONIC DIARRHŒA, SEQUEL OF TYPHOID FEVER.—This patient is a middle aged man who was admitted into the hospital six or seven weeks since, then in the third week of a severe enteric typhoid fever. He then presented all the phenomena of the gravest form of typhoid fever in its advanced stage, with more than the usual abdominal tympanites and intestinal looseness. The discharges from the bowels were not only very frequent and thin, but were stained with blood, and contained some mucus. He was sustained by regular use of wheat flour and milk porridge for nourishment, and the emulsion of oil of turpentine and tincture of opium every four hours, alternated with small doses of strychnia and nitric acid. In about one week after admission the symptoms of general fever disappeared, and the patient, though extremely weak, appeared convalescent in all respects, except the continuance of diarrhœa. His passages numbered from six to twelve per day, and continued thin, reddish-brown and offensive. Regarding this diarrhœa as dependent on the ulceration of the patches of aggregated glands, his diet was carefully regulated, and a variety of remedies, such as sub-nitrate of bismuth, carbolic acid, acetate of lead, and the mineral acids, each combined more or less with opiates, but with no other effect than temporary diminution of the number of intestinal discharges. In the meantime, two weeks had passed since his convalescence from the general fever, and his condition was very unpromising. There was much emaciation; haggard expression; quick, weak pulse; much abdominal tympanites and distention; and still from eight to twelve intestinal evacuations of thin, reddish-brown stools, often distinctly mixed with blood. He was then put on the use of syrup of ipecac and tincture of opium, equal parts mixed, half a fluid drachm every two hours. The dose was subsequently increased to nearly a drachm. During the first four or five days this produced a decidedly favorable influence, lessening the number of discharges, and improving their quality. After that time it began to lose its beneficial effect, and it was continued at intervals of every four hours, and a pill of nitrate of silver, one-third of a grain, with pulverized opium, two grains, was given between—making them two hours apart. From that time he began very gradually to improve, and after about two weeks his discharges were reduced to one in the twenty-four hours, but it was still thin. The interval between the doses of medicine was lengthened to six hours, at which rate he has continued to the present time, December 30, 1871.

He is now able to sit up a part of the day; is gaining in flesh; appetite good; but feet become œdematous when dependent. The Professor explained that the patient, though steadily improving, could not be left safely without further treatment. The ulcerated patches in the mucous membrane were not entirely cicatrized, and if treatment is discontinued too soon the diarrhœa will increase again. The patient was directed to adhere to a bland nutritious diet; take one of the pills of opium and nitrate of silver before breakfast, dinner and supper, and two-thirds of a teaspoonful of the mixture of syrup of ipecac and tincture of opium at bed time.

NOTE.—Jan. 30, 1872.—At this date the patient is apparently well, bowels regular and passages natural, suffering only from general debility. He has taken no medicine for the last week.

CASE V. ERYSIPELAS FOLLOWED BY DELIRIUM.—This case of erysipelas was of medium severity, with considerable œdema about jaw and neck—the inflammation having extended over the face and scalp. He was kept on Tinct. Ferri 20 drops every two hours with a local wash to relieve discomfort. He complained of being cold—probably due to impoverishment of blood. To support the circulation he had quinine gr. iiss, and Dover's powder gr. v, three times a day for two days. At the end of six days the inflammation was receding, when he became rather suddenly delirious. Bromide of potassium, in 15 gr. doses every three hours for two days, served to subdue the cerebral excitement. Because of his weak and depressed condition he then had quinine gr. iii, three times a day, with moderate doses of Tinct. Ferri, and has gradually recovered. The scabs and scales of exfoliating cuticle can be much earlier removed by softening with glycerine and washing with alkaline solutions.

